# Maternal Infection with *Trypanosoma cruzi* and Congenital Chagas Disease Induce a Trend to a Type 1 Polarization of Infant Immune Responses to Vaccines

**DOI:** 10.1371/journal.pntd.0000571

**Published:** 2009-12-22

**Authors:** Nicolas Dauby, Cristina Alonso-Vega, Eduardo Suarez, Amilcar Flores, Emmanuel Hermann, Marisol Córdova, Tatiana Tellez, Faustino Torrico, Carine Truyens, Yves Carlier

**Affiliations:** 1 Laboratoire de Parasitologie, Faculté de Médecine, Université Libre de Bruxelles (ULB), Brussels, Belgium; 2 Facultad de Medicina, Universidad Mayor de San Simon (UMSS), Cochabamba, Bolivia; Massachusetts General Hospital, United States of America

## Abstract

**Background:**

We previously showed that newborns congenitally infected with *Trypanosoma cruzi* (M+B+) display a strong type 1 parasite-specific T cell immune response, whereas uninfected newborns from *T. cruzi*-infected mothers (M+B−) are prone to produce higher levels of proinflammatory cytokines than control neonates (M−B−). The purpose of the present study was to determine if such fetal/neonatal immunological environments could alter the response to standard vaccines administered in early life.

**Methodology:**

Infants (6–7 months old) living in Bolivia, an area highly endemic for *T. cruzi* infection, and having received Bacillus Calmette Guerin (BCG), hepatitis B virus (HBV), diphtheria and tetanus vaccines, were enrolled into the M+B+, M+B−, M−B− groups mentioned above. The production of IFN-γ and IL-13, as markers of Th1 and Th2 responses respectively, by peripherical blood mononuclear cells stimulated with tuberculin purified protein derivative of *Mycobacterium tuberculosis* (PPD) or the vaccinal antigens HBs, diphtheria toxoid (DT) or tetanus toxoid (TT), as well as circulating levels of IgG antibodies against HBsAg, DT and TT were analyzed in infants. Cellular responses to the superantigen SEB were also monitored in M+B+, M+B−, M−B−infants and newborns.

**Principal Findings:**

M+B+ infants developed a stronger IFN-γ response to hepatitis B, diphtheria and tetanus vaccines than did M+B− and M−B− groups. They also displayed an enhanced antibody production to HBsAg. This was associated with a type 1-biased immune environment at birth, since cells of M+B+ newborns produced higher IFN-γ levels in response to SEB. M+B− infants produced more IFN-γ in response to PPD than the other groups. IL-13 production remained low and similar in all the three groups, whatever the subject's ages or vaccine status.

**Conclusion:**

These results show that: i) both maternal infection with *T. cruzi* and congenital Chagas disease do not interfere with responses to BCG, hepatitis B, diphtheria and tetanus vaccines in the neonatal period, and ii) the overcoming of immunological immaturity by *T. cruzi* infection in early life is not limited to the development of parasite-specific immune responses, but also tends to favour type 1 immune responses to vaccinal antigens.

## Author Summary

Vaccines are of crucial importance to prevent morbidity and mortality due to infectious diseases in childhood. A modulation of the fetal/neonatal immune system (considered immature) toward Th1 or Th2 dominance could modify responses to vaccines administered in early life. *T. cruzi* is the agent of Chagas' disease, in Latin America currently infecting about 2 million women at fertile ages who are susceptible to transmitting the parasite to their fetus. In previous studies we showed that *T. cruzi*-infected mothers can induce a pro-inflammatory environment in their uninfected neonates (M+B−), whereas congenitally infected newborns (M+B+) are able to develop a pro-Th1 parasite-specific T cell response. In the present study, we analysed the cellular and/or antibody responses to Bacillus Calmette Guerin (BCG), hepatitis B birus (HBV), diphtheria and tetanus vaccines in 6- to 7-month-old infants living in Bolivia. M+B− infants produced more IFN-γ in response to BCG, whereas M+B+ infants developed a stronger IFN-γ response to hepatitis B, diphtheria and tetanus vaccines and enhanced antibody production to HBs antigen. These results show that both maternal infection with *T. cruzi* and congenital Chagas disease do not interfere with responses to BCG, hepatitis B, diphtheria and tetanus vaccines in the neonatal period and that *T. cruzi* infection in early life tends to favour type 1 immune responses to vaccinal antigens.

## Introduction

Infectious diseases are a leading world-wide cause of morbidity and mortality in childhood, against which vaccination remains the best prevention measure [1]. However, protection induced by vaccines is of limited effectiveness in early life owing to the relative immaturity of the neonatal immune system. Moreover, the fetal/neonatal immune system is initially polarized toward a Th2 immune environment which appears essential for the survival of the fetus [2],[3]. Indeed, both dendritic cells and T cells present quantitative and qualitative defects in the neonatal period, limiting the development of CD4+ Th1 cell responses essential for the control of intra-cellular pathogens [2],[3], as well as the production of antibody responses [4]. Nonetheless, neonates are in some cases able to develop mature T cell responses. This has been demonstrated in congenital infections with *Trypanosoma cruzi* [5] and cytomegalovirus (CMV) [6], in infection with *Bordetella pertussis* in early life [7], and after early vaccinations with *Bacillus Calmette-Guerin* (BCG) [8] or the whole cell pertussis vaccine [9],[10]. Additionally, BCG vaccination at birth has been shown to increase both cellular and humoral responses to other vaccines such as hepatitis B and poliomyelitis vaccines [10]. Active maternal infections may also modulate neonatal immune responses to vaccines, as demonstrated in newborns of mothers chronically infected with helminths, who developed a Th2-biased response to BCG vaccination, by contrast with those born to non-infected mothers [11],[12]. The modulation of immune responses to vaccines in infants from mothers infected with intracellular parasites, and having experienced such congenital infection has heretofore not been investigated.

Chagas disease, or American trypanosomiasis, caused by the protozoan parasite *Trypanosoma cruzi*, is a major cause of morbidity and mortality in Latin America where 8–10 million people are currently estimated to be infected [13]. It has also become an important health issue in the United States and Europe due to large-scale migration of Latin Americans over the last few decades [14]. The parasite is primarily transmitted by insect vectors (in endemic areas of Latin America), blood transfusion and congenitally (in all areas) [15]. Even the frequency of transmission by vectors and blood transfusion decreases as a result of vectorial control programmes and improvement of blood bank screening, mother-to-child transmission of *T. cruzi* presently cannot be prevented and has thus become an important route of transmission [16]. Recent estimations indicate that at least 15,000 newborns are likely to be congenitally infected with *T. cruzi* each year in Latin America [17] and 2,000 in North America [18]. In Europe, such transmission also becomes a problem in migrants originating from endemic countries [19]–[21]. In Bolivia, a highly endemic area for Chagas disease, we have reported that 17% of pregnant women are chronically infected with *T. cruzi* and that congenital transmission occurs in 5 to 6% of the cases [22]. We have showed that congenitally infected newborns develop a parasite-specific T cell immune response comparable to that of adults [5] as well as phenotypic and functional modifications of their NK cells [23]. On the other hand, newborns of *T. cruzi-*infected mothers are prone to produce higher levels of pro-inflammatory cytokines in comparison to those born to non-infected mothers [24]. The present study aimed to determine if such modifications of the immune environment in infected or uninfected newborns of *T. cruzi*-infected mothers could modulate immune responses to vaccines administered at birth or in early life. Cellular and/or humoral responses to BCG, hepatitis B virus (HBV), diphtheria and tetanus vaccines were compared in infants living in Bolivia.

## Methods

### Patients and diagnosis of *T. cruzi* infection

Two different patient groups from Cochabamba (Bolivia) were enrolled in this study: one of neonates (born at the German Urquidi Maternity) and one of infants (6 to 7 months old; followed at the Manuel Ascenci Villaroel paediatric Hospital and the Viedma Universitary Hospital, UMSS). The scientific/ethic committee of the Universidad Mayor de San Simon (UMSS) approved the study, and informed written consent was obtained from the mothers before blood collection. Maternal *T. cruzi* infection was assessed by standard serological techniques. *T. cruzi* infection in neonates and infants was diagnosed by detection of parasites in umbilical cord or peripheral blood by microscopic examination of heparinized microhematocrit tubes, or in some cases by hemoculture, as previously described [16],[22]. Congenital infection was assessed at or close to birth for 28 neonates and 10 infants. For 3 other infants, *T. cruzi* infection was diagnosed later on when they presented at 6–7 months old at the consultation. Congenital infection was inferred because these infants lived in an area free of vectorial transmission, had not travelled in endemic areas, and had not received blood transfusion.

Three groups of neonates and infants were established: congenitally-infected (M+B+); non-infected born to *T. cruzi*-infected mothers (M+B−); and uninfected controls born to uninfected mothers (M−B−). A total of 68 newborns and 60 infants were included in the study (M+B+: 28 newborns and 13 infants; M+B−: 19 newborns and 36 infants; M−B−: 21 newborns and 11 infants).

Mothers of neonates and infants included in the study were all asymptotic, even when infected with *T. cruzi*. They originate from the same rural region surrounding Cochabamba and live in similar socioeconomic environment. The sex ratio in all groups of newborns or infants was similar (data not shown). Clinical data of the newborn groups have been previously reported [22] and Table 1 shows mean ages and weights at the time of blood collection, as well as historical clinical data at birth, of newborn and infant groups. At the time of blood collection, the infant age in the 3 groups was comparable and M+B− and M−B− infants were all asymptomatic. At birth, such infants had similar APGAR scores and maturity parameters. However, M+B+ infants presented at birth slightly lower mean gestational age, weight and APGAR scores; three of them showed one or two symptoms known to be associated with congenital *T. cruzi* infection (i.e., splenomegaly, anasarca and petechia). When examined at 6 to 7 months, M+B+ infants were asymptomatic, but had still slightly lower mean weight as compared to the other infants.

**Table 1:** Characterization of the cohorts of neonates and infants.

Variable (mean±SD)	M−B−	M+B−	M+B+
**Neonates** a			
N	21	19	28
APGAR 1 min	7.9±0.5	8.2±0.6	7.7±1.5
APGAR 5 min	9.1±0.8	9.3±0.6	9.3±1.5 (p = 0.059)^c^
Gestational age	39.7±0.5	39.7±1.12	38.9±2.2 (p = 0.052)^c^
Birth weight (g)	3329±355	3367±305	3073±385 (p = 0.003)^c^
**Infants** a			
N	11	36	13
Age (days)^b^	196±21	192±17	195±14
APGAR 1 min	8.0±0.2	8.0±0.4	7.4±1.6
APGAR 5 min	9.3±0.5	9.4±0.5	8.9±0.9 (p = 0.076)^c^
Gestational age at birth	39.8±0.5	39.4±0.9	38.9±0.8 (p = 0.044)^c^
Birth weight (g)	3338±335	3171±436	2971±459 (p = 0.072)^c^
Actual weight (g)^b^	8160±576	8258±936	7757±928 (p = 0.060)^c^

**M+**: *T. cruzi* infected mothers; **M−**: not infected mothers; **B+**: congenitally infected with *T. cruzi*; **B−**: not infected.

a Number of newborns or infants per group are indicated in Methods.

b At the time of blood sampling.

c The value of p compares congenital subjects to the 2 other groups together (Mann-Withney *U* test) and is given when <0.1.

### Management of patients

As soon as the diagnosis of congenital *T. cruzi* infection was established, treatment of infected neonates/infants (7–10 mg/kg/day of benznidazole for 30 days, [22]) was proposed to the mother. From the 13 M+B+ infants under study, 9 were treated within the two first months after birth, and 4 remained untreated at 6–7 months of age when blood was collected for the present immunological study. One remained untreated since her mother refused the treatment, and 3 were treated from 6–7 months old onwards since the diagnosis of infection was not established previously (see above). Cure was confirmed in treated cases by the subsequent negative results of direct parasitological examination (microhematocrit tubes) and *T. cruzi* serology. All M−B− and M+B− infants presented with negative serology for *T. cruzi* at 6 to 7 months of age, indicating that they had not acquired the infection after birth.

### Vaccination programme

In accordance with the recommended Bolivian vaccination programme, all newborns received the BCG vaccine (BCG, Serum Institute of India Ltd) at birth by intradermic injection. Infants were then vaccinated against diphtheria, tetanus, whooping cough, hepatitis B and type B *Haemophilus influenzae* (Hib) by receiving intramuscular injections of Tritanrix mixed with Hiberix (GlaxoSmithkline Biologicals, Rixensart, Belgium) at the ages of 2, 4 and 6 months. At the same time, they also received the oral vaccine against poliomyelitis (attenuated vaccine, trivalent OPV, Sanofi Pasteur, Paris, France). Infants included in this study have all received the BCG at birth. At the time of blood sampling, 53.5% of the infants had completed the vaccinal schedule (3 doses of Tritanrix + Hiberix + OPV), the others having received 2 doses. The proportion of infants having received the 3 doses was similar in each group.

### Collection of blood samples, cell isolation and in vitro cultures

Cord blood (CB) and infant peripheral blood (PB) were collected in sterile endotoxin-free heparinized tubes. Plasma was stored at −20°C until use for determinations of antibody levels. Mononuclear cells (CBMC and PBMC) were immediately isolated by Nycoprep density gradient centrifugation (Nycomed Pharma AS, Oslo, Norway). CBMC and PBMC (2×10^6^ cells/mL - duplicates) were then cultured in RPMI containing 10% fetal calf serum (Biowhittaker, Lonza, Verviers, Belgium), either alone to measure spontaneous production of cytokines, or in the presence of one of the following antigens: tuberculin purified protein derivative of *M. tuberculosis* (PPD; 5 µg/mL; batch RT49, Statens Seruminstitut, Denmark), diphtheria toxoid (DT; 10 µg/mL), tetanus toxoid (TT; 10 µg/mL), or the surface S antigen of HBV (HbsAg; 10 µg/mL). DT, TT and HbsAg were kindly provided by GlaxoSmithkline Biologicals (Rixensart, Belgium). A positive control for T lymphocyte stimulation was included by incubating cells with the staphylococcal enterotoxin B (SEB; 10 ng/mL; Sigma-Aldrich) activating non specifically T cells (superantigen). After 6 days of culture at 37°C in 5% CO_2_ humidified air, cell cultures were centrifuged and supernatants conserved at −70°C for further cytokine analysis.

### Cytokine determinations

IFN-γ and IL-13 cytokine concentrations were used as markers of Th1/Th2 responses, respectively. Cytokine levels were measured in culture supernatants by ELISA using commercial kits (IFN-γ: antibody pairs and standard from Biosource, Invitrogen, Merelbeke, Belgium; IL-13: ultrasensitive kit from Bender Medsystems, VWR International, Leuven, Belgium). Standards were non-glycosylated recombinant cytokines (Biosource and Bender Medsystems). Detection limits were 2 pg/mL for IFN-γ, and 1 pg/mL for IL-13. Spontaneous release of cytokines by unstimulated cells was generally undetectable or very low. In the latter case, cytokine concentration was subtracted from levels obtained after stimulation with SEB or vaccine antigens.

### HbsAg, TT and DT-specific antibody determinations

IgG antibodies against HbsAg, DT and TT were measured in plasma samples by ELISA using commercial kits (HbsAg: Diasorin, Brussels, Belgium, DT and TT: Genzyme Virotech GmbH, Rüsselsheim, Germany). Results in international units (IU) were derived from WHO standard sera provided with the kits. Antibody concentrations above 10 IU/mL for hepatitis B, and 0.1 IU/mL for diphtheria and tetanus were considered to be protective [25]–[27].

IgG sub-classes against HbsAg were measured by ELISA as follow : Nunc Maxisorp plates (VWR) were coated with HbsAg (GSK Biologicals) at a concentration of 1.5 µg/mL in PBS overnight at 4°C. After blocking with 1% bovine serum albumin (BSA) in PBS for 2h at 37°C, plasma samples were diluted in PBS containing 0.3% BSA and 0.05% Tween 20 and incubated for 2h at 37°C. Sample dilutions were 1/50 for IgG1 and 1/10 for other IgG sub-classes. Mouse monoclonal antibodies specific for the Fc fragment of each human IgG subclass (clones HP6001, HP6014, I7260 and HP6050 for anti-IgG1, G2, G3 and G4 antibodies respectively, Sigma-Aldrich, Bornem, Belgium) were diluted 1/500, and then incubated for 1h at 37°C, followed by rat monoclonal antibodies specific for mouse IgG coupled to horseradish peroxidase (clone LO-MK-1, Imex, UCL, Brussels, Belgium) at 1 µg/mL for 1h at 37°C. After each step, plates were washed with PBS containing 0.05% Tween 20. Finally, substrate and chromogen were added (hydrogen peroxide and 3,3′, 5,5′ tétraméthylbenzidine, BD Biosciences, Erembodegem-Aalst, Belgium) and absorbance at 450 nm was measured after 30 min colour development and stopping the reaction with 2N sulphuric acid. Positive and negative internal controls were added in each ELISA plate in order to correct the absorbances for plate variability. We found a very good correlation between the levels of total HbsAg-specific IgG measured with this ELISA and those obtained using the commercial Diasorin kit (r = 0.842, p<10^−4^, n = 25). Absorbances lower than 0.01 were considered a negative result.

### Expression of results and statistical analysis

Results were expressed either as arithmetic means±SEM or geometric means. Statistical comparisons of concentrations of cytokine or antibody levels between groups of newborns or infants were performed either with the Mann Whitney test or by two-way analysis of variance (ANOVA), followed by Tukey test for multiple comparisons. Comparisons of proportions were carried out using the Fisher exact test. Statistical analyses were conducted using GraphPad Prism software (GraphPad Prism 4, San Diego, CA) or the Statistical Software Package SPSS version17.0.

## Results

### Congenitally *T. cruzi*-infected newborns display a polarized Th1 response to SEB

**Figure 1:**
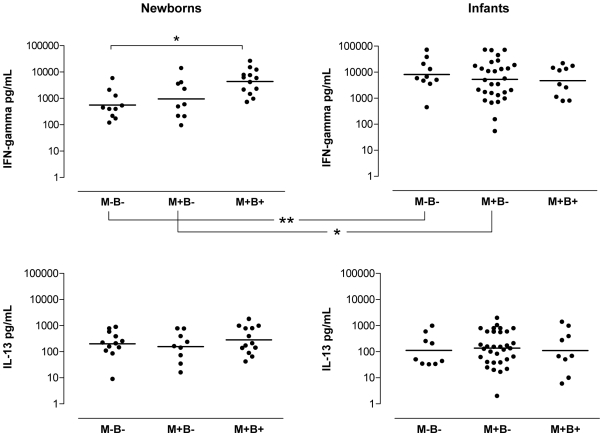
IFN-γ and IL-13 productions in response to SEB by blood mononuclear cells from newborns and infants. Newborns and 6 to 7 months-old infants were born either to *T. cruzi-*infected (M+) or not infected mothers (M−), and either congenitally infected (B+) or not (B−). Cells were cultured during 6 days in the presence of 10 ng/mL SEB (staphylococcal enterotoxin B). Results of cytokines levels measured by ELISA (individual results and geometric means) are shown. * : p<0.05 and ** : p<0.005 (Mann-Withney *U* test).

Fig. 1 shows that, upon SEB stimulation, cells from M+B+ newborns produced significantly more IFN-γ in comparison with the M−B− neonate group (geometric means 4372 and 551 pg/mL respectively). Interestingly, such congenitally infected neonates displayed at birth comparable levels to those observed in controls at 6–7 months (geometric means in M−B− infants : 4726 pg/mL). Meanwhile, IL-13 levels remained similar in all neonate groups. Comparable results were seen in the M+B− and M−B− groups. This indicates that the *in utero* immune environment associated with congenital Chagas disease induces an early shift in the neonatal immune response toward a type 1 response.

### Congenitally *T. cruzi*-infected infants present a Th1 shifted response to vaccines against diphtheria, tetanus and HBV

IFN-γ and IL-13 were not detected at birth when CBMC were incubated with DT, TT and HbsAg vaccinal antigens. However, as shown in Table 2, these cytokines were detected in 6 to 7 month old infants. Two way Anova followed by a test for multiple comparisons (Tukey) indicated that the IFN-γ responses in the different infant groups was not influenced by the used antigen (p>0.05), allowing us to compare the responses after globalizing the results of each antigen. This showed IFN-γ responses to vaccinal antigens to be significantly higher in congenitally-infected M+B+ infants as compared to each other groups (M+B+ vs. M−B− : p = 0.007 ; M+B+ vs. M+B− : p = 0.0003) whereas they were similar between M−B− and M+B− infants (p>0.05). Thus, M+B+ infants produced meanly 10–12 times more IFN-γ to HbS and DT, and 3–4 times more of this cytokine in response to TT. IL-13 release to these 3 vaccinal antigens did not differ between groups. This highlights a trend towards a type 1 response for vaccines against hepatitis, diphtheria and tetanus administered in congenitally infected neonates. Since the benznidazole treatment received by M+B+ infants before vaccination might have some confounding effect on stimulating immune responses [28], data were also analyzed separating treated and untreated infants (at the time of blood collection). Similar results were observed in both M+B+ subgroups (data not shown).

**Table 2:** Production of IFN-γ and IL-13 in response to vaccinal antigens by PBMC from infants from mothers infected (M+) or not (M−),with *T. cruzi*, and congenitally infected (B+) or not (B−).

Vaccinal Ag	Infant group	IFN-γ (pg/mL)^a^	IL-13 (pg/mL)^a^
**HBsAg**	M−B−	22.2±2.6	10.1±4.6
	M+B−	18.5±6.3	37.2±31.1
	M+B+	221±151	19.2±17
**Diphtheria toxoid**	M−B−	27.3±0.4	6.2±0.5
	M+B−	40.5±24.6	59.9±51.4
	M+B+	354±158	12.9±8.8
**Tetanus toxoid**	M−B−	58.3±24.7	8.4±6.0
	M+B−	107±42	20.9±6.7
	M+B+	205±94	20.5±11.4

a Arithmetic mean±SEM.

### Congenitally *T. cruzi*-infected infants produce higher antibody levels against HbsAg in response to HBV vaccination

**Figure 2:**
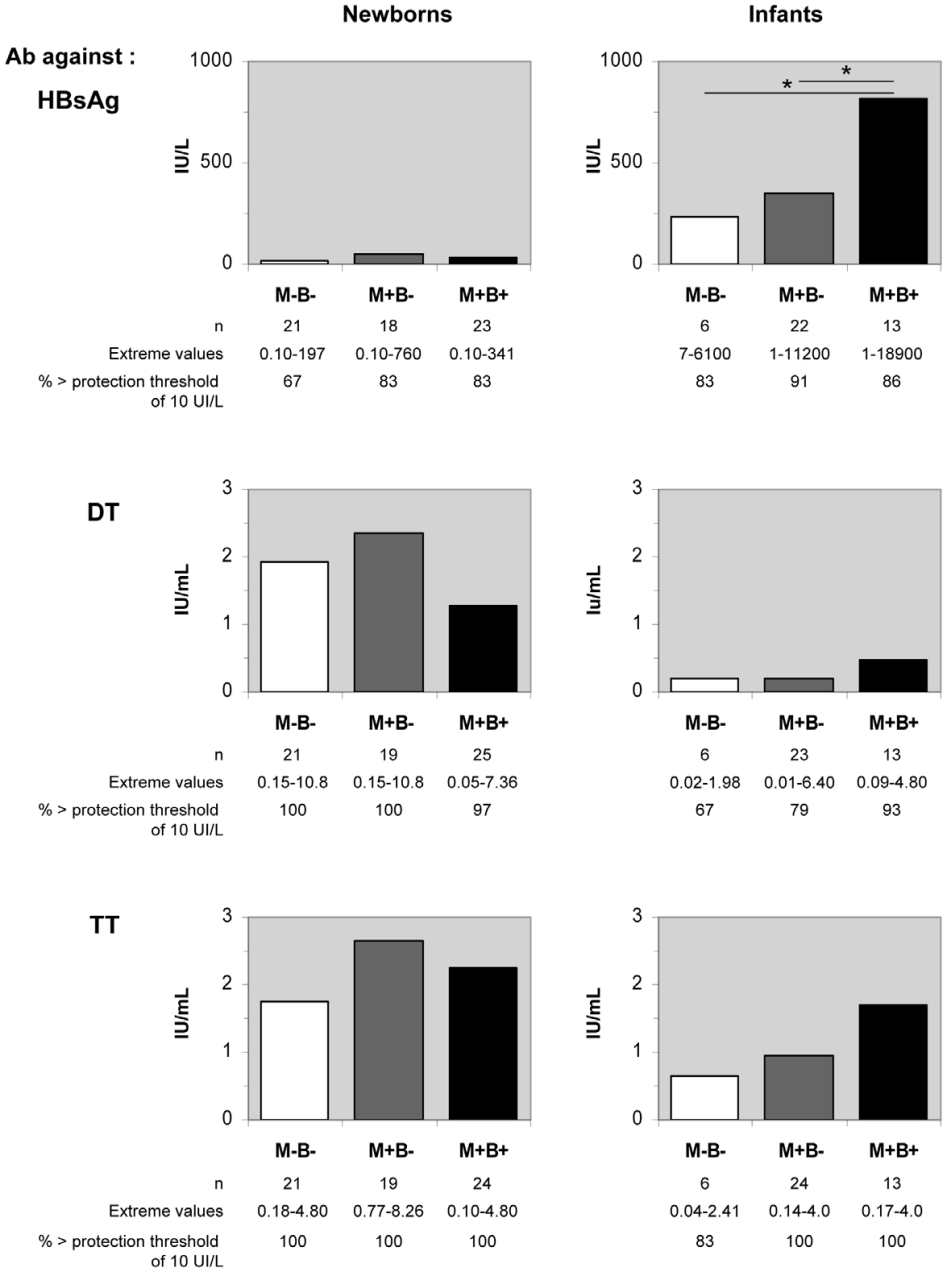
Antibody levels against hepatitis B, diphtheria and tetanus vaccines in newborns and infants. Plasma levels of specific IgG in newborns and 6 to 7 months old infants born to mothers infected (M+) or not (M−) with *T. cruzi*, and congenitally infected (B+) or not (B−), and vaccinated at 2, 4 and 6 months against hepatitis B, diphtheria and tetanus. Results are shown as geometric means; extreme values are indicated, as well as the proportion of samples showing an antibody level above the indicated threshold of protection. * : p<0.05 (Mann Withney *U* test).

As indicated in Fig. 2, mean levels of IgG antibodies against the vaccinal antigens HbsAg, DT, TT at birth were similar in the 3 groups of neonates, and high enough to be considered protective (67 to 100% above the protection thresholds of 10 IU/mL for hepatitis B, and 0.1 IU/mL for diphtheria and tetanus). This indicates that mothers had been immunized and that the transfer of such maternal antibodies during pregnancy was not affected by *T. cruzi* infection.

In the 3 infant groups, HbsAg-specific antibody levels were significantly higher than in newborns. This sustains HbsAg-specific antibodies to be produced by the infants in response to vaccination, although maternally transmitted antibodies may still be present at low levels their sera [29]. Inversely, DT- and TT-specific antibody levels were lower in infants than at birth, suggesting that maternally transmitted antibodies may have restricted their production [30]. Nevertheless, they are close to antibody levels described by others in vaccinated infants [31]. Interestingly, M+B+ infants produced noticeably more antibody in response to the vaccine against hepatitis than those of the other groups. Though not statistically significant, a similar trend was seen for the M+B+ DT- and TT IgG antibodies. For the reasons mentioned above, M+B+ HbsAg-specific antibody levels were also analyzed by separating treated and untreated infants, and, again, results were similar in both M+B+ subgroups (data not shown).

**Figure 3:**
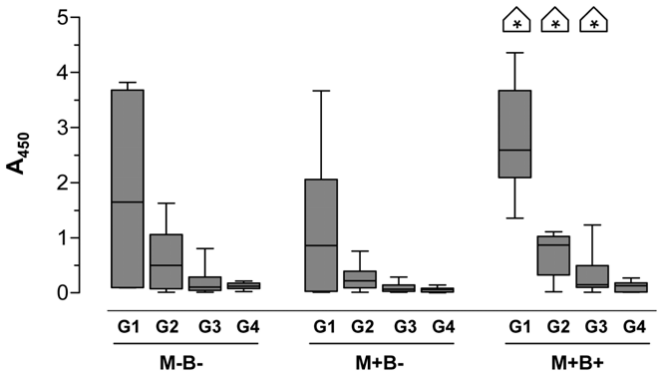
Levels of antibody IgG sub-classes against hepatitis B in vaccinated infants. Plasma levels of HbsAg-specific IgG sub-classes in 6 to 7 months old infants born to mothers infected (M+) or not (M−) with *T. cruzi*, and congenitally infected (B+) or not (B−), and vaccinated at 2, 4 and 6 months against hepatitis B. Results are displayed as box and whiskers (n = 6 M−B−, 13 M+B− and 9 M+B+). * : p<0.05 compared with infants from other groups (Man Withney *U* test).

Since the polarization of the immune response is known to modulate the isotypes of antibodies produced [32]–[34], we next determined the IgG subclass profile of the HbsAg-specific antibodies in vaccinated M+B+ infants. As shown in Fig. 3 and as expected from previous reports [35],[36], IgG1 was the main subclass of IgG antibody found in infants vaccinated against HBV regardless of the group. Comparisons between groups show that M+B+ infants harboured 2 to 4-fold higher levels of IgG1, IgG2 and IgG3 than infants of the other groups, while IgG4 levels remained low in all groups. Such variations likely account for the greater production of total HbsAg-specific IgG described above in these M+B+ infants, likely reflecting their stronger type 1 immune environment.

### Uninfected infants born to *T. cruzi*-infected mothers display an amplified type 1 response to PPD after BCG vaccination

**Figure 4:**
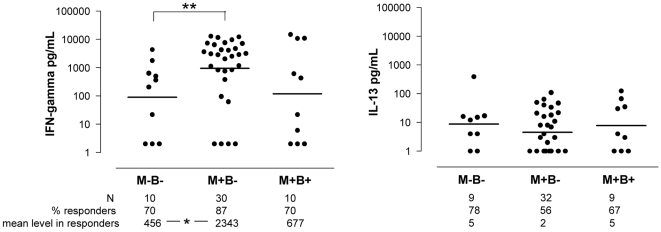
Cytokine response to PPD in infants vaccinated with BCG. IFN-γ and IL-13 production in response to tuberculin purified protein derivative of *M. tuberculosis* (PPD) by PBMC from 6 to 7 months old infants born to mothers infected (M+) or not (M−) with *T. cruzi*, and congenitally infected (B+) or not (B−), and having received at birth the BCG vaccine. PBMC were cultured during 6 days in the presence of 10 µg/mL PPD. Results of cytokines levels measured by ELISA are shown (individual results and geometric means). The proportions of responders (i.e. showing detectable levels of cytokines) are also shown. * : p<0.05 and ** : p = 0.006 compared with M−B− infants (Mann-Withney *U* test for means and Fisher test for proportions).

We also studied the Th1/Th2 cytokine responses to the BCG vaccine by stimulating mononuclear cells with mycobacterial PPD. CBMC (newborns) did not produce either IFN-γ or IL-13 after PPD stimulation, regardless of group (a total of 32 newborns were tested). By contrast, PBMC from most infants having received the BCG vaccine at birth produced cytokines in response to PPD (Fig. 4). The proportions of IFN-γ responders was ≥70% and similar in the 3 infant groups. However, M+B− but not M+B+ infants, produced on average 5-fold more IFN-γ in response to PPD in comparison to control M−B− infants. IL-13 levels produced by M+B− infants in response to PPD were low, and the proportion of IL-13 responders was slightly but significantly inferior to that of both other groups. This suggests that, even in the absence of congenital *T. cruzi* transmission, the maternal-fetal immune environment associated with maternal infection contributes to enhance the Th1 response to the BCG vaccine administered at birth.

## Discussion

We have investigated the effect of congenital Chagas disease and/or maternal *T. cruzi* infection on immune responses to standard vaccines regularly administered in neonates and infants in Bolivia. Our results show that: i) congenitally *T. cruzi*-infected newborns (M+B+) display an early ability to produce the type 1 cytokine IFN-γ; ii) their specific responses (M+B+) to the hepatitis B vaccine, and to a lesser extend to diphtheria and tetanus vaccines, administered later after birth are characterized by higher levels of IFN-γ and/or protective IgG antibodies; and iii) non-infected infants born to *T. cruzi*–infected mothers (M+B−) secrete higher amounts of IFN-γ in response to BCG vaccination administered at birth.

The responses to PPD that we observed in BCG-vaccinated infants were Th1-oriented in the 3 studied groups. This agrees with previous observations showing that BCG vaccine given at birth induces a strong Th1 response that modulates the neonatal Th2 immune environment, with the CD4+ subpopulation of T cells being the main source of IFN-γ [8],[37]. However, M+B− infants mounted a still stronger Th1 response to BCG. This likely relates to the prenatal activation of the immune system induced by maternal *T. cruzi* infection described by us and others, leading to a non-specific activation of various cell types as monocytes, lymphocytes and probably other cells [24],[38]. Such activation might result from the transplacental transfer of soluble molecules released by parasites present in the infected mother, such as the Tc52 molecule known to activate directly dendritic cells or pro-inflammatory glycophosphatidyl-inositol anchors [39]–[41]. Our study shows that chronic maternal infection with an intracellular pathogen can influence the neonatal immune environment sufficiently to enhance the Th1 regular infant response to BCG. This is also in line with other work showing, conversely, that neonates born to mothers chronically infected by helminths, known to promote Th2 immune responses, display diminished responses to PPD [11],[12],[42].

By contrast, M+B+ infants failed to exhibit an increased response to BCG vaccine. This is surprising, since they presented at birth, when BCG was administered, a marked type 1 immune bias (indicated by their increased IFN-γ response to SEB), and harboured high amounts of circulating live parasites [43], susceptible to release the pro-inflammatory molecules mentioned above. Different hypotheses could explain such divergence between M+B− and M+B+ infant responses to BCG. First, the treatment received by M+B+ infants might have contributed to modify their response; the similar PPD responses observed in treated and untreated infants may not rule out a treatment effect since the number of cases in each subgroup was low. The M+B+ IFN-γ−producing T cells might also have been temporary exhausted as indicated by their high level of apoptosis resulting from their intense response to parasite multiplication [5], subsequently limiting the responses to other antigens, as also shown in other infections [44],[45]. Another possibility relates to the higher frequency of prematurity and low birth weights in the M+B+ newborn group [22], known to reinforce immune immaturity [46],[47]. Furthermore, congenital *T. cruzi* infection might have down-regulated the cord blood T cell response, as observed in malaria [48]. Finally, it is also possible that the IFN-γ response to BCG increases earlier in M+B+ infants than in other groups, and is no more detected at 6–7 months, in agreement with the observation that there is no more difference in the response to SEB between groups at 6–7 months.

Nevertheless, the immune responses of M+B+ infants to hepatitis B vaccine, and to a lesser extend against diphtheria and tetanus vaccines, given after birth, were stronger and more type 1-oriented than in both of the other groups of uninfected infants. This group produced significantly higher specific IgG levels in response to HBV vaccine. A similar trend was observed for antibody responses to diphtheria and tetanus vaccines. Enhanced antibody responses in M+B+ infants were associated with increased IFN-γ production in response to vaccine antigens. The observation that, amongst the HbsAg-specific IgG, the IgG1, IgG2 and IgG3 subclasses, known to be associated with type 1-responses to intracellular pathogens [32]–[34] were increased still emphasizes the Th1-biased profile of the response. The mechanism of such type 1-shift of the immune response to DT, TT and HBV vaccines associated with congenital Chagas disease, and why it is not observed in M+B− infants, remains to be elucidated. Some non-exclusive factors might be considered. First, the fact that the immunological modulation observed at birth in M+B− newborns is transient [38] could imply that it could have a short term impact on immune responses to vaccines given from 2 months of age onwards. Secondly, the benznidazole treatment received by M+B+ infants might release parasitic pro-inflammatory molecules (see above) from lysed parasites favouring IFN-γ production [28]. However, high parasite levels were no longer present at the time of diphtheria, tetanus and HBV vaccine administration (2, 4 and 6 months). No differences in the IgG and IFN-γ responses to DT, TT and HBV antigens were observed between treated and untreated infants, suggesting that treatment has a minimal effect on the Th1 shift observed in M+B+ infants. Third, the type of vaccine might also play a role on the different responses between M+B+ and M+B− infants. Indeed, BCG is a cellular, live attenuated vaccine, meaning that non virulent mycobacterias multiply in the host receiving the vaccine, which induces mainly a T cell response (CD4+ Th1 and CD8+), whereas the vaccines against hepatitis B, diphtheria and tetanus are acellular vaccines eliciting mainly a B cell production of IgG antibodies with the help of T cells. Our results suggest that at birth, the immune environment in uninfected newborns from *T. cruzi*-infected mothers (M+B−) favours preferentially a cellular response, while that of congenitally-infected infants (M+B+) is more prone to modulate antibody responses.

If the increased competence to secrete IFN-γ in both M+B− and M+B+ infants might confer better or earlier protection against infection may be discussed. Indeed, our study was limited to blood samples collected from infants at 6–7 months of age. Earlier sample collection would be useful for kinetic studies to appreciate a possible earlier protective effect. A follow-up might also permit to compare the functional implication of the protective effect of the different vaccinations between infant groups. Though the response to PPD does not correlate with protection against tuberculosis [49], we may assume that it reflects a globally better response to BCG, a vaccine known to limit the severity of tuberculosis if acquired during early infancy, although it does not prevent the acquisition of infection [50]. For the other vaccines, the fact that M+B+ infants produce higher amounts of protective antibodies suggests that protection is probably reached earlier in life in infants suffering from congenital Chagas disease.

Although this study involved a limited number of cases, our results show that both maternal infection with *T. cruzi* and congenital Chagas disease have no major impediment on vaccinal responses to BCG, hepatitis B, diphtheria and tetanus vaccines in the neonatal period. Nevertheless, both conditions induced a trend towards a type 1 immune polarization known to be essential in fighting intracellular pathogens [3]. In addition, our results also show that the overcoming of immunological immaturity by *T cruzi* infection in early life is not limited to the development of parasite-specific immune response [5], but has also effects on responses to parasite-unrelated vaccinal antigens. This suggests adjuvant properties of some *T. cruzi* molecules, offering new prospects in neonatal vaccinology.

## References

[1] WHO (2005).

[2] Marchant A, Goldman M (2005). T cell-mediated immune responses in human newborns: ready to learn?. Clin Exp Immunol.

[3] Seder RA, Hill AV (2000). Vaccines against intracellular infections requiring cellular immunity.. Nature.

[4] Siegrist CA (2007). The challenges of vaccine responses in early life: selected examples.. J Comp Pathol.

[5] Hermann E, Truyens C, Alonso-Vega C, Even J, Rodriguez P (2002). Human fetuses are able to mount an adultlike CD8 T-cell response.. Blood.

[6] Marchant A, Appay V, Van Der SM, Dulphy N, Liesnard C (2003). Mature CD8(+) T lymphocyte response to viral infection during fetal life.. J Clin Invest.

[7] Mascart F, Verscheure V, Malfroot A, Hainaut M, Pierard D (2003). *Bordetella pertussis* infection in 2-month-old infants promotes type 1 T cell responses.. J Immunol.

[8] Vekemans J, Amedei A, Ota MO, D'Elios MM, Goetghebuer T (2001). Neonatal bacillus Calmette-Guerin vaccination induces adult-like IFN-gamma production by CD4+ T lymphocytes.. Eur J Immunol.

[9] Mascart F, Hainaut M, Peltier A, Verscheure V, Levy J (2007). Modulation of the infant immune responses by the first pertussis vaccine administrations.. Vaccine.

[10] Ota MO, Vekemans J, Schlegel-Haueter SE, Fielding K, Sanneh M (2002). Influence of *Mycobacterium bovis* bacillus Calmette-Guerin on antibody and cytokine responses to human neonatal vaccination.. J Immunol.

[11] Malhotra I, Mungai P, Wamachi A, Kioko J, Ouma JH (1999). Helminth- and Bacillus Calmette-Guerin-induced immunity in children sensitized in utero to filariasis and schistosomiasis.. J Immunol.

[12] Labeaud AD, Malhotra I, King MJ, King CL, King CH (2009). Do antenatal parasite infections devalue childhood vaccination?. PLoS Negl Trop Dis.

[13] Schofield CJ, Jannin J, Salvatella R (2006). The future of Chagas disease control.. Trends Parasitol.

[14] Schmunis GA (2007). Epidemiology of Chagas disease in non-endemic countries: the role of international migration.. Mem Inst Oswaldo Cruz.

[15] Carlier Y, Pinto Dias JC, Ostermayer Luquetti AO, Hontebeyrie M, Torrico F, Editions scientifiques et médicales Elsevier SAS, editors. (2002). Trypanosomiase américaine ou maladie de Chagas.. Encycl Méd Chir,Maladies Infectieuses.

[16] Carlier Y, Torrico F (2003). Congenital infection with *Trypanosoma cruzi*: from mechanisms of transmission to strategies for diagnosis and control.. Revista da Sociedade Brasileira de Medicina Tropical.

[17] Jannin J, Salvatella R (2006). Estimación Cuantitativa de la Enfermedad de Chagas en las Américas - Quantitative estimates of Chagas disease in the Americas.

[18] Buekens P, Almendares O, Carlier Y, Dumonteil E, Eberhard M (2008). Mother-to-child transmission of Chagas' disease in North America: why don't we do more?. Matern Child Health J.

[19] Riera C, Guarro A, Kassab HE, Jorba JM, Castro M (2006). Congenital transmission of *Trypanosoma cruzi* in Europe (Spain): a case report.. Am J Trop Med Hyg.

[20] Munoz J, Portus M, Corachan M, Fumado V, Gascon J (2007). Congenital *Trypanosoma cruzi* infection in a non-endemic area.. Trans R Soc Trop Med Hyg.

[21] Jackson Y, Myers C, Diana A, Marti HP, Wolff H (2009). Congenital transmission of chagas disease in latin american immigrants in Switzerland.. Emerg Infect Dis.

[22] Torrico F, Alonso-Vega C, Suarez E, Rodriguez P, Torrico MC (2004). Maternal *Trypanosoma cruzi* infection, pregnancy outcome, morbidity, and mortality of congenitally infected and non-infected newborns in Bolivia.. Am J Trop Med Hyg.

[23] Hermann E, Alonso-Vega C, Berthe A, Truyens C, Flores A (2006). Human congenital infection with *Trypanosoma cruzi* induces phenotypic and functional modifications of cord blood NK cells.. Pediatr Res.

[24] Vekemans J, Truyens C, Torrico F, Solano M, Torrico MC (2000). Maternal *Trypanosoma cruzi* infection upregulates capacity of uninfected neonate cells To produce pro- and anti-inflammatory cytokines.. Infect Immun.

[25] Hasselhorn HM, Nubling M, Tiller FW, Hofmann F (1998). Factors influencing immunity against diphtheria in adults.. Vaccine.

[26] Simonsen O, Bentzon MW, Heron I (1986). ELISA for the routine determination of antitoxic immunity to tetanus.. J Biol Stand.

[27] West DJ, Calandra GB (1996). Vaccine induced immunologic memory for hepatitis B surface antigen: implications for policy on booster vaccination.. Vaccine.

[28] Sathler-Avelar R, Vitelli-Avelar DM, Massara RL, de Lana M, Pinto Dias JC (2008). Etiological treatment during early chronic indeterminate Chagas disease incites an activated status on innate and adaptive immunity associated with a type 1-modulated cytokine pattern.. Microbes Infect.

[29] Caceres VM, Strebel PM, Sutter RW (2000). Factors determining prevalence of maternal antibody to measles virus throughout infancy: a review.. Clin Infect Dis.

[30] Siegrist CA (2003). Mechanisms by which maternal antibodies influence infant vaccine responses: review of hypotheses and definition of main determinants.. Vaccine.

[31] Saffar MJ, Khalilian AR, Ajami A, Saffar H, Qaheri A (2008). Seroimmunity to diphtheria and tetanus among mother-infant pairs; the role of maternal immunity on infant immune response to diphtheria-tetanus vaccination.. Swiss Med Wkly.

[32] Avery DT, Bryant VL, Ma CS, de Waal MR, Tangye SG (2008). IL-21-induced isotype switching to IgG and IgA by human naive B cells is differentially regulated by IL-4.. J Immunol.

[33] Snapper CM, Paul WE (1987). Interferon-gamma and B cell stimulatory factor-1 reciprocally regulate Ig isotype production.. Science.

[34] Garraud O, Perraut R, Riveau G, Nutman TB (2003). Class and subclass selection in parasite-specific antibody responses.. Trends Parasitol.

[35] Gregorek H, Madalinski K, Woynarowski M, Mikolajewicz J, Syczewska M (2000). The IgG subclass profile of anti-HBs response in vaccinated children and children seroconverted after natural infection.. Vaccine.

[36] Rossi ME, Azzari C, Resti M, Appendino C, Pezzati P (1998). Selectivity in IgG subclass response to hepatitis B vaccine in infants born to HBsAg-positive mothers.. Clin Exp Immunol.

[37] Murray RA, Mansoor N, Harbacheuski R, Soler J, Davids V (2006). Bacillus Calmette Guerin vaccination of human newborns induces a specific, functional CD8+ T cell response.. J Immunol.

[38] Neves SF, Eloi-Santos S, Ramos R, Rigueirinho S, Gazzinelli G (1999). In utero sensitization in Chagas' disease leads to altered lymphocyte phenotypic patterns in the newborn cord blood mononuclear cells.. Parasite Immunol.

[39] Ouaissi A, Guilvard E, Delneste Y, Caron G, Magistrelli G (2002). The *Trypanosoma cruzi* Tc52-released protein induces human dendritic cell maturation, signals via Toll-like receptor 2, and confers protection against lethal infection.. J Immunol.

[40] Almeida IC, Gazzinelli RT (2001). Proinflammatory activity of glycosylphosphatidylinositol anchors derived from *Trypanosoma cruzi*: structural and functional analyses.. J Leukoc Biol.

[41] Acosta-Serrano A, Almeida IC, Freitas-Junior LH, Yoshida N, Schenkman S (2001). The mucin-like glycoprotein super-family of *Trypanosoma cruzi*: structure and biological roles.. Mol Biochem Parasitol.

[42] Carlier Y, Truyens C (1995). Influence of maternal infection on offspring resistance towards parasites.. Parasitol Today.

[43] Virreira M, Truyens C, Alonso-Vega C, Brutus L, Jijena J (2007). Comparison of *Trypanosoma cruzi* lineages and levels of parasitic DNA in infected mothers and their newborns.. Am J Trop Med Hyg.

[44] Kasprowicz V, Schulze Zur WJ, Kuntzen T, Nolan BE, Longworth S (2008). High level of PD-1 expression on hepatitis C virus (HCV)-specific CD8+ and CD4+ T cells during acute HCV infection, irrespective of clinical outcome.. J Virol.

[45] Blackburn SD, Wherry EJ (2007). IL-10, T cell exhaustion and viral persistence.. Trends Microbiol.

[46] Sadeghi K, Berger A, Langgartner M, Prusa AR, Hayde M (2007). Immaturity of infection control in preterm and term newborns is associated with impaired toll-like receptor signaling.. J Infect Dis.

[47] Kaur K, Chowdhury S, Greenspan NS, Schreiber JR (2007). Decreased expression of tumor necrosis factor family receptors involved in humoral immune responses in preterm neonates.. Blood.

[48] Brustoski K, Moller U, Kramer M, Hartgers FC, Kremsner PG (2006). Reduced cord blood immune effector-cell responsiveness mediated by CD4+ cells induced in utero as a consequence of placental *Plasmodium falciparum* infection.. J Infect Dis.

[49] Elias D, Akuffo H, Britton S (2005). PPD induced in vitro interferon gamma production is not a reliable correlate of protection against *Mycobacterium tuberculosis*.. Trans R Soc Trop Med Hyg.

[50] Doherty TM, Andersen P (2005). Vaccines for tuberculosis: novel concepts and recent progress.. Clin Microbiol Rev.

